# Clinical features and outcomes of in-hospital cardiac arrest in code blue events: a retrospective observational study

**DOI:** 10.3389/fcvm.2023.1247340

**Published:** 2023-11-06

**Authors:** Masayuki Akatsuka, Hiroomi Tatsumi, Yoshiki Masuda

**Affiliations:** Department of Intensive Care Medicine, School of Medicine, Sapporo Medical University, Sapporo, Japan

**Keywords:** in-hospital cardiac arrest, outcome, clinical features, CASPRI score, retrospective study

## Abstract

**Background:**

In-hospital cardiac arrest (IHCA) is a critical medical event with outcomes less researched compared to out-of-hospital cardiac arrest. This retrospective observational study aimed to investigate key aspects of IHCA epidemiology and prognosis in patients with Code Blue activation.

**Methods:**

This retrospective observational study enrolled patients with Code Blue events in our hospital between January 2010 and October 2019. Participant characteristics, including age and sex, and IHCA characteristics, including the time of cardiac arrest, witnessed event, bystander cardiopulmonary resuscitation (CPR), initial shockable rhythm, vital signs at 1 and 6 h before IHCA, survival to hospital discharge (SHD), and the cardiac arrest survival postresuscitation in-hospital (CASPRI) score were included in univariate and multivariate logistic regression analyses with SHD as the primary endpoint.

**Results:**

From the 293 Code Blue events that were activated during the study period, 81 participants were enrolled. Overall, the SHD rate was 28.4%, the median CPR duration was 14 (interquartile range, 6–28) min, and the rate of initial shockable rhythm was 19.8%. There were significant intergroup differences between the SHD and non-SHD groups in the CPR duration, shockable rhythm, and CASPRI score on univariate logistic regression analysis. Multivariate logistic regression analysis showed that the CASPRI score was the most accurate predictive factor for SHD (OR = 0.98, *p *= 0.006).

**Conclusions:**

The CASPRI score is associated with SHD in patients with IHCA during Code Blue events. Therefore, the CASPRI score of IHCA patients potentially constitutes a simple, useful adjunctive tool for the management of post-cardiac arrest syndrome.

## 1. Introduction

Cardiac arrests can occur in diverse settings, including hospitals, homes, and public spaces (1–3). Cardiac arrest that occurs within the hospital environment is termed in-hospital cardiac arrest (IHCA) and is a potentially fatal event that has an incidence of 3–6 of 1,000 hospitalizations (4). Despite constituting a significant public health problem, IHCA has received less research and public awareness attention than out-of-hospital cardiac arrest (OHCA). Survival rates for IHCA vary widely, with approximately 24% of IHCA patients surviving to hospital discharge. However, of those who survive, 14% experience neurological disability (5). This highlights the importance of early recognition and appropriate management of IHCA to improve survival outcomes and minimize neurological damage. Furthermore, despite the importance of IHCA, there is a lack of information on the evidence and clinical features of IHCA compared to OHCA.

Research to bridge this knowledge gap has involved the investigation of factors associated with IHCA outcomes (6–9), and some studies evaluated the Cardiac Arrest Survival Postresuscitation In-hospital (CASPRI) score, which was developed to predict the outcomes of IHCA patients (10, 11). The CASPRI score is calculated based on several clinical variables, including age, initial rhythm, prearrest Cerebral Performance Category (CPC) score, hospital unit where the cardiac arrest occurred, and duration of resuscitation (10). The CASPRI score is a reliable predictor of IHCA outcomes, with accuracy rates ranging from 70% to 90% (12, 13).

Despite an increasing understanding of IHCA, compared to OHCA, there is a paucity of information on the clinical features and outcomes of IHCA. This study was conducted with an aim to address this knowledge gap by clarifying the clinical characteristics of IHCA and investigating predictive factors of IHCA patients with Code Blue events.

## 2. Materials and methods

### 2.1. Study design and ethics approval

In this retrospective observational study, we enrolled patients with Code Blue events that occurred in our hospital between January 2010 and October 2019. Participants were categorized into two groups: those who survived to hospital discharge (SHD) and those who did not (non-SHD). We conducted intragroup comparisons to identify key characteristics associated with survival to hospital discharge. This study was conducted in accordance with the Strengthening the Reporting of Observational Studies in Epidemiology (STROBE) guidelines and was approved by the Institutional Review Board of Sapporo Medical University (authorization number 312–175).

### 2.2. Participants

Patients with Code Blue events were identified from the hospital patient information system. Code Blue was defined as an emergency call activated for cases with strongly suspected IHCA, comprising sudden loss of consciousness and respiratory arrest. Patients with do-not-attempt-resuscitation (DNAR) orders were excluded from the analysis.

### 2.3. Data collection

For all participants who met the inclusion criteria, we collected detailed demographic, clinical, and laboratory data from the electronic medical records. All data were de-identified prior to analysis.

### 2.4. Statistical analysis

Categorical variables are expressed as numbers and percentages, and continuous variables as mean with standard deviation or median with interquartile range (IQR), as appropriate. The Cardiac Arrest Survival Postresuscitation In-hospital (CASPRI) score, a composite score, was calculated based on several clinical variables, including age, initial rhythm, prearrest CPC score, hospital unit where the cardiac arrest occurred, and duration of resuscitation. The categorical and continuous variables were analyzed using the chi-square and Mann–Whitney *U*-tests, respectively. Statistical analyses were performed using IBM SPSS Statistics version 27 (IBM, Armonk, NY, USA). A *p*-value <0.05 was considered statistically significant. We conducted univariate and multivariate regression analyses to identify the factors associated with survival to hospital discharge (SHD).

## 3. Results

**Figure 1:**
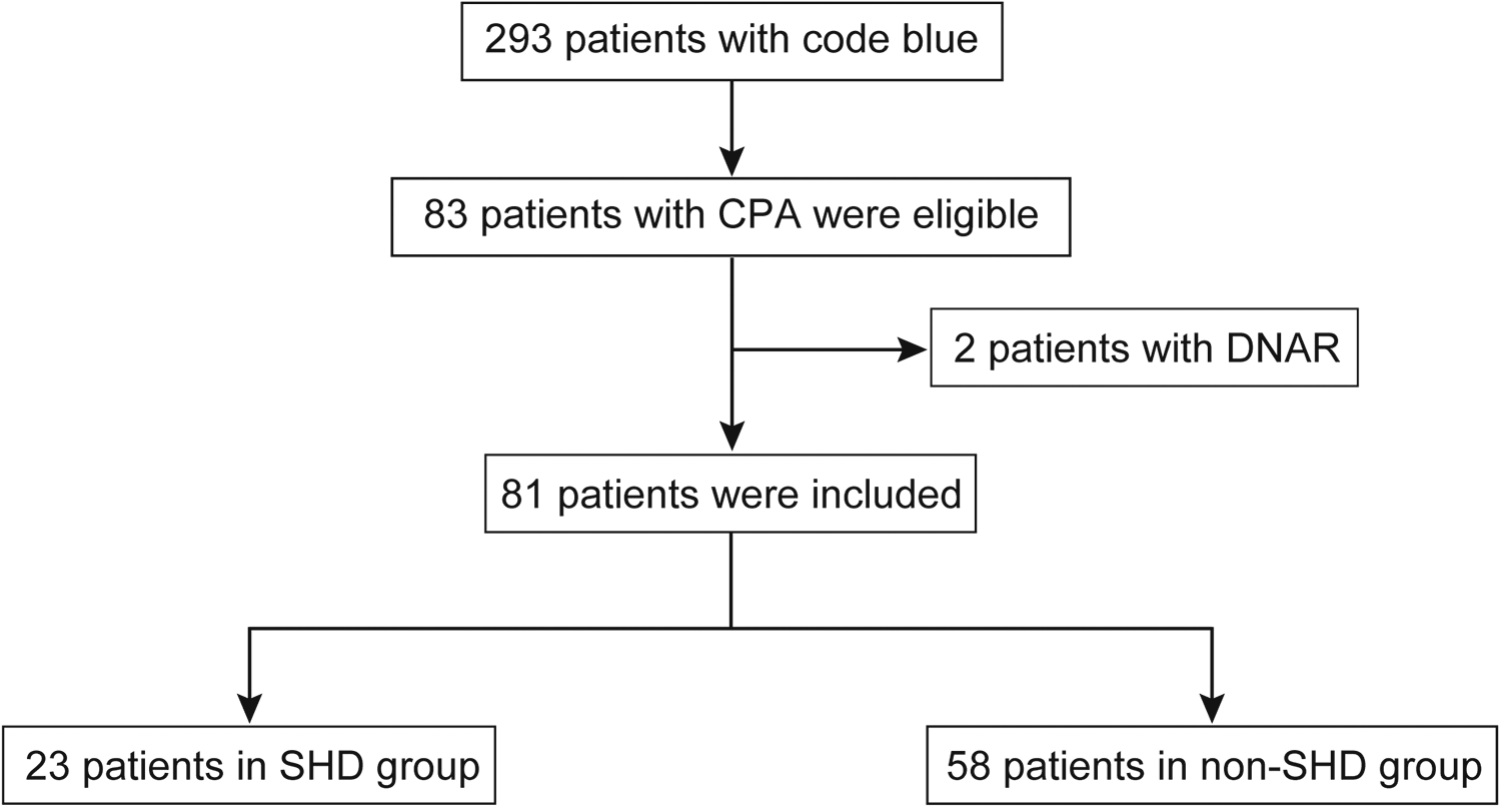
Flow diagram of this study. CPA, cardiopulmonary arrest; DNAR, do not attempt resuscitation; SHD, survival to hospital discharge.

Details of participant selection and enrollment are shown in Figure 1. Of the 293 patients who experienced Code Blue events, 83 had an IHCA. After excluding 2 patients with DNAR orders, 81 participants were included in our study. Of these 81 participants, 23 (28.4%) survived to hospital discharge whereas 58 (71.6%) did not.

### 3.1. Participant demographic

There were no significant intergroup differences in age, sex, presence of witnessed onset, bystander cardiopulmonary resuscitation (CPR), or time of day of cardiac arrest between the SHD and non-SHD groups (Table 1). The most common location of cardiac arrest was the hospital room for both groups (78.3% and 81.0%, respectively). There was no significant intergroup difference for the time of day when the cardiac arrest occurred (*p* = 0.073) or in tracheal intubation, signs at 1 or 6 h before cardiac arrest, or shockable rhythm. Compared to the non-SHD group, the CPR duration was significantly shorter (*p *< 0.001) and the CASPRI score was significantly lower (*p *< 0.001) in the SHD group.

**Table 1:** Comparing the baseline characteristics of in-hospital cardiac arrest cases based on the SHD.

Variable	SHD		*p*
	Yes (*n* = 23)	No (*n* = 58)	
Age (years)	70 (66–77)	70 (62–79)	0.99
Male, *n* (%)	15 (65.2)	37 (63.8)	0.90
Cardiac arrest during the day time, *n* (%)	15 (65.2)	25 (43.1)	0.073
Witnessed, *n* (%)	20 (87.0)	43 (74.1)	0.21
Bystander CPR, *n* (%)	23 (100)	55 (94.8)	0.55
CPR duration (minutes)	6 (4–13)	18 (10–33)	<0.001
Location, *n* (%)			
Hospital room	18 (78.3)	47 (81.0)	
CT room	2 (8.7)	4 (6.9)	
MRI room	1 (4.4)	0 (0)	
Examination room	1 (4.4)	1 (1.7)	
Entrance to the hospital	0 (0)	3 (5.2)	
Rest room	0 (0)	1 (1.7)	
Others	1 (4.4)	2 (3.4)	
Intubation, *n* (%)	16 (69.6)	48 (82.8)	0.23
Shockable rhythm, *n* (%)	8 (34.8)	8 (13.8)	0.06
Signs 1 h before arrest, *n* (%)	10 (43.5)	32 (55.2)	0.34
Signs 6 h before arrest, *n* (%)	7 (30.4)	19 (32.8)	0.84
CASPRI score	16 (12–20)	23.5 (20–26)	<0.001

Data are presented as median (IQR) or frequency (percentage).

SHD, survival to hospital discharge; CPR, cardiopulmonary resuscitation; CASPRI score, cardiac arrest survival postresuscitation in-hospital score.

### 3.2. Univariate regression analysis

We found significant differences in the CPR duration and shockable rhythm between the SHD and non-SHD group (odds ratio [OR], 0.93; 95% confidence interval [CI], 0.88–0.98; *p* = 0.005 and OR, 0.30; 95% CI, 0.10–0.94; *p* = 0.038, respectively). There were no significant intergroup differences in other variables (Table 2).

**Table 2:** Predictors of survival to hospital discharge (SHD) in in-hospital cardiac arrest based on univariate and multivariate analyses.

Variable	Univariate		Multivariate	
	OR (95% CI)	*p*	OR (95% CI)	*p*
Age	1.00 (0.96–1.04)	0.91	–	–
Sex	0.94 (0.34–2.58)	0.90	–	–
Arrest time	0.40 (0.15–1.10)	0.077	–	–
CPR duration	0.93 (0.88–0.98)	0.005	0.96 (0.91–1.02)	0.16
Witnessed	0.75 (0.14–4.08)	0.74	–	–
Intubation	2.10 (0.69–6.43)	0.19	–	–
Shockable rhythm	0.30 (0.10–0.94)	0.038	1.67 (0.32–8.57)	0.54
Signs 1 h before arrest	1.60 (0.60–4.24)	0.34	–	–
Signs 6 h before arrest	1.11 (0.39–3.16)	0.84	–	–
CASPRI score	0.82 (0.74–0.91)	<0.001	0.83 (0.73–0.95)	0.006

OR, odds ratio; CI, confidence interval; CPR, cardiopulmonary resuscitation.

### 3.3. Multivariate regression analysis

Multivariate regression analysis was performed with the CPR duration, shockable rhythm, and CASPRI score as explanatory variables. The CASPRI score was the most significant predictive factor for SHD (OR, 0.83; 95% CI, 0.73–0.95; *p* = 0.006; Table 2).

## 4. Discussion

We assessed the factors associated with SHD in patients with IHCA. The CASPRI score was the most accurate SHD predictor in a tertiary hospital in a Japanese population. Our study provides valuable insight into the contributory factors for the survival of IHCA patients. This is in line with previous research that has demonstrated the utility of the CASPRI score for predicting outcomes in IHCA patients (12, 13).

Our study found that the CASPRI score was the most effective predictor of SHD, and this indicates that the risk stratification of patients based on the abovementioned factors can help healthcare providers identify those who may benefit from more aggressive resuscitative interventions. This finding is particularly important, given that IHCA is associated with high mortality rates, and the identification of predictors of survival can help improve patient outcomes. Additionally, the results of univariate regression analysis showed that a shorter CPR duration and the presence of shockable rhythm are associated with higher SHD rates. These findings are consistent with the results of previous research (1, 12, 14), indicating that effective and prompt resuscitative interventions could improve the likelihood of favorable outcomes in patients with IHCA. The above-discussed results highlight the importance of high-quality CPR and rapid defibrillation for improving patient outcomes following IHCA.

Compared to previous studies, this study found some differences in the predictors of SHD in patients with IHCA. Specifically, this study found that age, sex, and comorbidities were not significantly associated with survival, which contradicts the findings of some previous studies that found these factors to be predictors of survival (6, 15–17). A possible explanation for these discrepancies is the difference in the study populations. This study was conducted in a tertiary hospital in Japan, whereas previous studies have included patients from diverse settings and geographic locations. Therefore, it is possible that patient characteristics, resuscitation protocols, and other factors may vary between hospitals and regions, which could modulate the association between the predictors and the outcomes.

Our study did not find significant associations between the type and severity of the underlying disease and survival to hospital discharge. This finding suggests that the CASPRI score, along with other factors like CPR duration and the presence of a shockable rhythm, may play a more crucial role in predicting outcomes in patients with IHCA. The absence of a significant association between underlying disease and survival highlights the need for a multifactorial approach to risk stratification and resuscitative interventions in IHCA cases, irrespective of the type or severity of the underlying disease.

Overall, this study provides valuable insight into the predictors of SHD in patients with IHCA, particularly in a Japanese population. Although some of our findings are similar to those of some previous studies, there are differences that may be attributed to differences in the study populations, resuscitation protocols, and other factors. Continuing to identify predictors of outcomes in this patient population can help improve the effectiveness of resuscitative interventions and thereby enhance patient care.

The findings of this study have practical implications for improving the survival of patients with IHCA. The identification of the CASPRI score as a reliable predictor for survival can aid healthcare providers in risk stratification, allowing them to identify patients who may benefit from more aggressive resuscitative interventions. Additionally, the results underscore the importance of high-quality cardiopulmonary resuscitation (CPR) and prompt defibrillation in IHCA cases. To improve IHCA outcomes, a multifaceted approach that includes risk stratification, early intervention, and adherence to established protocols is essential.

While our study focused on IHCA, it raises the interesting question of whether there are commonalities or distinctions in factors predicting survival between IHCA and OHCA. Future research that compares and contrasts these two settings may provide valuable insights into the management of cardiac arrest across different environments.

One of the strengths of our study is that it adds to the existing literature on the utility of the CASPRI score in predicting the outcomes in patients with IHCA. Furthermore, this study identified additional factors, such as the presence of shockable rhythm and shorter CPR duration, that may contribute to the survival of patients with IHCA. These findings highlight the importance of prompt and effective resuscitation interventions for improving patient outcomes.

Our study has several limitations that need consideration. It was conducted at a single research center, which may limit the generalizability of the findings to other settings and populations. The retrospective nature of the study also presents a limitation, and prospective research would be valuable in confirming and extending our results. Furthermore, our research did not specifically examine the effect of other interventions, such as therapeutic hypothermia or advanced airway management, which have been previously identified as predictors of IHCA outcomes. The small sample size and the fact that the SHD group was only half the size of the non-SHD group are other important limitations.

In conclusion, this study provides valuable insights into the factors associated with SHD in patients with IHCA. The CASPRI score, along with a shorter CPR duration and the presence of a shockable rhythm, emerged as significant predictors of SHD. Risk stratification based on these factors can help healthcare providers identify patients who may benefit from more aggressive resuscitation interventions, ultimately improving patient care. Further research is needed to validate these findings and identify additional predictors of outcomes in this patient population.

## Data Availability

The original contributions presented in the study are included in the article/Supplementary Material, further inquiries can be directed to the corresponding author.
